# Abrogation of self-tolerance by misfolded self-antigens complexed with MHC class II molecules

**DOI:** 10.1126/sciadv.abj9867

**Published:** 2022-03-04

**Authors:** Hui Jin, Kazuki Kishida, Noriko Arase, Sumiko Matsuoka, Wataru Nakai, Masako Kohyama, Tadahiro Suenaga, Ken Yamamoto, Takehiko Sasazuki, Hisashi Arase

**Affiliations:** 1 Laboratory of Immunochemistry, WPI Immunology Frontier Research Center, Osaka University, Suita City, Osaka 565-0871, Japan.; 2 Department of Immunochemistry, Research Institute for Microbial Diseases, Osaka University, Suita City, Osaka 565-0871, Japan.; 3 Department of Dermatology, Osaka University Graduate School of Medicine, Suita City, Osaka 565-0871, Japan.; 4 Department of Microbiology, Fukushima Medical University, Fukushima City, Fukushima 960-1295, Japan.; 5 Department of Medical Biochemistry, Kurume University School of Medicine, Kurume City, Fukuoka 830-0011, Japan.; 6 Kyushu University Institute for Advanced Study, Fukuoka City, Fukuoka 812-8582, Japan.

## Abstract

Specific MHC class II alleles are strongly associated with susceptibility to various autoimmune diseases. Although the primary function of MHC class II molecules is to present peptides to helper T cells, MHC class II molecules also function like a chaperone to transport misfolded intracellular proteins to the cell surface. In this study, we found that autoantibodies in patients with Graves’ disease preferentially recognize thyroid-stimulating hormone receptor (TSHR) complexed with MHC class II molecules of Graves’ disease risk alleles, suggesting that the aberrant TSHR transported by MHC class II molecules is the target of autoantibodies produced in Graves’ disease. Mice injected with cells expressing mouse TSHR complexed with MHC class II molecules, but not TSHR alone, produced anti-TSHR autoantibodies. These findings suggested that aberrant self-antigens transported by MHC class II molecules exhibit antigenic properties that differ from normal self-antigens and abrogate self-tolerance, providing a novel mechanism for autoimmunity.

## Teaser

The TSH receptor/MHC class II complex is involved in Graves’ disease pathogenesis.

## INTRODUCTION

Graves’ disease (GD) is a common organ-specific autoimmune disease with an annual incidence of 20 to 50 cases per 100,000 persons (*1*). The primary symptoms are hyperthyroidism and goiter, with almost a half of GD patients reporting symptoms of Graves’ ophthalmopathy (*2*, *3*). Thyroid-stimulating hormone receptor (TSHR) is a major thyroid self-antigen, and autoantibodies against TSHR are widely believed to be the cause of hyperthyroidism (*4*). Autoantibodies that bind to specific epitopes on the TSHR mimic thyroid-stimulating hormone and induce the secretion of excessive amounts of thyroid hormone from thyroid cells (*2*).

Major histocompatibility complex (MHC) class II molecules play a central role in the immune response by presenting peptide antigens to helper T cells (*5*, *6*). In humans, MHC molecules are referred to as human leukocyte antigen (HLA). Although the expression of HLA class II molecules is restricted to macrophages, B cells, and dendritic cells in normal situations, it has been known that the aberrant HLA class II expression has been observed on thyroid follicular cells in patients with GD (*7*). On the other hand, specific HLA class II alleles have been reported to be associated with the risk of GD (*8*), with HLA-DPB1*05:01, in particular, being strongly associated with the development of GD in the Japanese population (*9*–*12*). Nevertheless, the mechanisms underlying autoantibody production in GD patients as well as in other autoimmune diseases have remained unclear. In addition, the mechanisms for the association of specific HLA class II alleles and the risk of GD are also unclear.

We have recently found a novel function of MHC class II molecules, that is, the transport of cellular misfolded proteins to the cell surface without processing them into peptides (*13*). Furthermore, self-antigens complexed with MHC class II molecules of autoimmune disease risk alleles exhibit antigenic properties different from those of normal antigens and are major autoantibody targets in several autoimmune diseases (*14*–*18*). Autoantibody binding to self-antigens complexed with MHC class II molecules is significantly associated with the risk of developing rheumatoid arthritis and anti-neutrophil cytoplasmic antibody (ANCA)–associated vasculitis, suggesting that self-antigens complexed with MHC class II molecules are involved in the etiology of autoimmune diseases (*14*, *16*). However, the pathophysiological function of self-antigen/MHC class II complexes in vivo has not yet been elucidated. In this study, we addressed the function of TSHR complexed with MHC class II molecules in recognition by GD autoantibodies ex vivo and in the induction of anti-TSHR autoantibodies in vivo.

## RESULTS

### Autoantibodies from GD patients bind to TSHR in the presence of MHC class II molecules with the GD risk allele

**Fig. 1.**
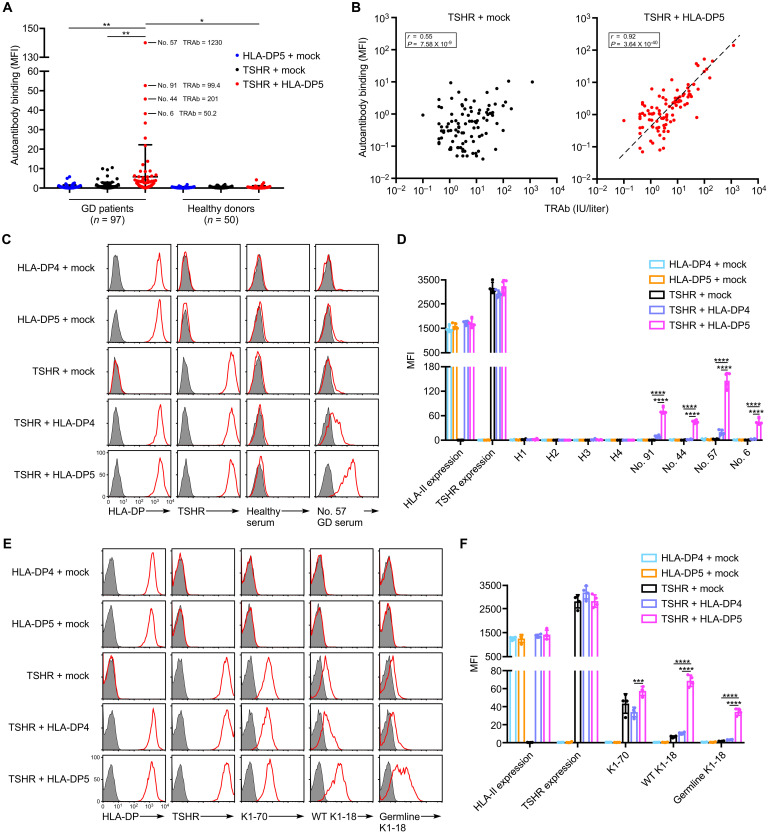
Human TSHR complexed with HLA-DP5 is recognized by autoantibodies from GD patients. (**A**) HLA-DP5, full-length human TSHR (TSHR), or TSHR with HLA-DP5 was transfected into HEK293T cells. The transfectants were mixed with sera from GD patients or healthy donors, and IgG Ab binding was detected. The MFIs of autoantibody binding are represented as dots (one per individual) with the mean ± SD, unpaired two-tailed Student’s *t* test. Four representative GD patients are indicated. (**B**) The MFIs for the binding of autoantibodies from GD patients’ sera to TSHR or TSHR with HLA-DP5 in (A) were plotted against the TRAb of each GD. Linear regression line (black dotted line), correlation coefficient (*r*), and *P* value for the regression line are shown. (**C**) HLA-DP4, HLA-DP5, TSHR, TSHR with HLA-DP4, or TSHR with HLA-DP5 was transfected into HEK293T cells. The transfectants were mixed with serum from the representative GD patient (no. 57) or a healthy donor, and IgG Ab binding was detected (red). The expression of HLA-DP and TSHR (red) is also shown. As a control, cells were stained with APC-conjugated anti-mouse or anti-human IgG Ab alone (shaded histograms). (**D**) The same transfectants used in (C) were mixed with sera from other healthy donors (H) or GD patients, and IgG Ab binding was detected. The MFIs for four independent cell stains are bar plotted with the mean ± SD, multiple *t* tests. (**E**) HLA-DP4, HLA-DP5, TSHR, TSHR with HLA-DP4, or TSHR with HLA-DP5 was transfected into HEK293T cells. The transfectants were stained with K1-70, wild-type (WT) K1-18, or germlined K1-18 mAb (red). The expression of HLA-DP and TSHR (red) is also shown. As a control, cells were stained with APC-conjugated anti-mouse or anti-human IgG Ab alone (shaded histograms). (**F**) Bar plots of (E); the MFIs for four independent cell stains are shown with the mean ± SD, multiple *t* tests. Mock: Empty pME18S plasmid. *P < 0.05, **P < 0.01, ***P < 0.001, and ****P < 0.0001. All data are representative of at least three independent experiments.

We analyzed the binding of autoantibodies from GD (table S1) and healthy donors against TSHR transfectants in the presence or absence of HLA-DP5, a GD risk allele (HLA-DPA1*02:02/HLA-DPB1*05:01). Some sera from GD patients, but not healthy donors, bound efficiently to TSHR in the presence of HLA-DP5. In contrast, only a few of sera from patients bound weakly to TSHR in the absence of HLA-DP5, although the expression levels of TSHR were almost the same (Fig. 1A). Thyroid-stimulating autoantibodies recognize specific epitopes on TSHR, and thyroid-stimulating hormone receptor antibody (TRAb) is usually measured by competitive binding assay using a thyroid-stimulating M22 monoclonal antibody (mAb) (third-generation assay) in clinical laboratory tests (*19*, *20*). The levels of autoantibodies bound to TSHR in the presence of HLA-DP5 were strongly correlated with TRAb titers (*r* = 0.92). However, there was no significant correlation in the absence of HLA-DP5 (*r* = 0.55) (Fig. 1B). This suggests that epitopes for thyroid-stimulating autoantibodies in GD patients are exposed on TSHR through the association with HLA-DP5.

We next compared the effect of HLA class II alleles on autoantibody binding to TSHR between HLA-DP5, a GD risk allele, and HLA-DP4, a GD-unassociated allele (HLA-DPA1*01:03/HLA-DPB1*04:01). Autoantibodies bound efficiently to TSHR in the presence of HLA-DP5 molecules but bound very weakly to TSHR in the presence of HLA-DP4 molecules, although the expression levels of TSHR were almost the same (Fig. 1, C and D). These data indicate that the HLA-DP5 molecule, a GD risk allele, is involved in the recognition of TSHR by autoantibodies from GD patients and that TSHR complexed with HLA-DP5 is a major target of GD autoantibodies.

### TRAb preferentially binds to TSHR complexed with MHC class II molecules

We investigated whether HLA-DP5 was involved in the recognition of TSHR by mAbs derived from GD patients. K1-18, a TRAb from a GD patient (*21*), bound more strongly to TSHR cotransfected with HLA-DP5 than to TSHR cotransfected with HLA-DP4 or without HLA class II molecules (Fig. 1, E and F). In contrast, HLA-DP5 did not affect the recognition of TSHR by the K1-70 monoclonal anti-TSHR–blocking autoantibody (Fig. 1, E and F) (*21*). This also suggests that epitopes for thyroid-stimulating autoantibodies are exposed on TSHR through the association with HLA-DP5.

Antigen-stimulated B cells acquire somatic mutations in the genes coding for the variable regions of the heavy and light chains during affinity maturation (*22*). In contrast, naive B cells do not have such mutations and, therefore, the variable regions are encoded by germline genes (*23*). Recently, Di Zenzo *et al.* (*24*) demonstrated that autoantibodies lose the ability to bind to self-antigens when their variable regions revert to the germline sequence, suggesting that autoantibodies appear to be induced by non–self-antigens. Accordingly, we generated a germlined K1-18 mAb that was mutated to the germline variable region sequence of the heavy and light chains. Although the binding of the germlined K1-18 was decreased by the mutation, the germlined K1-18 mAb still recognized TSHR in the presence of HLA-DP5, but not in the presence of HLA-DP4 or TSHR alone (Fig. 1, E and F). These findings demonstrate that TSHR complexed with HLA-DP5, but not HLA-DP4 or TSHR alone, represents an original antigen that induces TRAb.

### The TSHR ECD complexed with the peptide-binding groove of HLA-DP5 is the target of TRAb

**Fig. 2.**
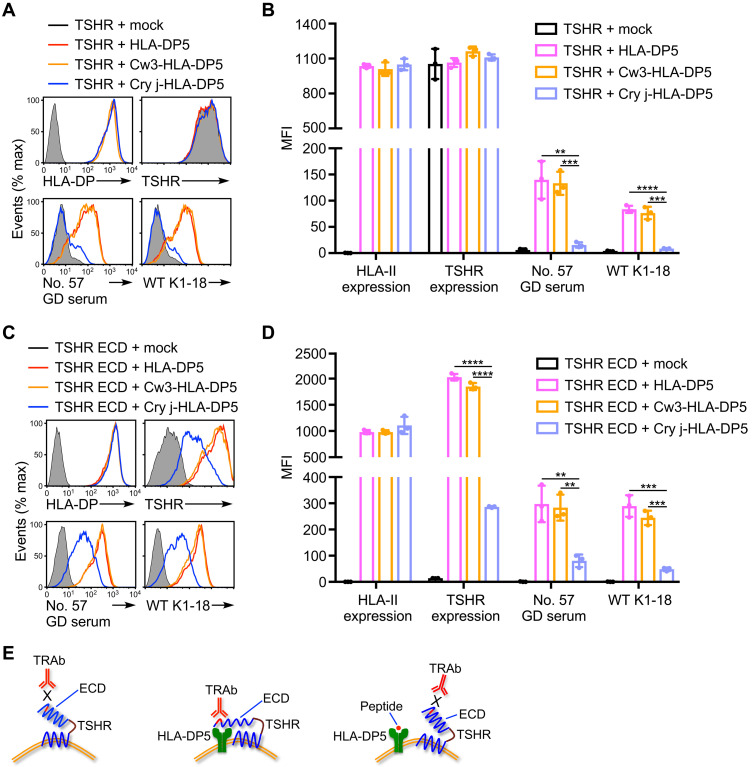
TSHR extracellular domain binds to the peptide-binding groove of HLA-DP5. (**A** and **C**) (A) Full-length human TSHR (TSHR) (shaded histograms), TSHR with HLA-DP5 (red lines), TSHR with HLA-Cw3 peptide–attached HLA-DP5 (Cw3-HLA-DP5, yellow lines), TSHR with Cry-j-1 pollen peptide–attached HLA-DP5 (Cry j-HLA-DP5, blue lines), (C) TSHR extracellular domain (ECD) (shaded histograms), TSHR ECD with HLA-DP5 (red lines), TSHR ECD with HLA-Cw3 peptide–attached HLA-DP5 (Cw3-HLA-DP5, yellow lines), or TSHR ECD with Cry-j-1 pollen peptide–attached HLA-DP5 (Cry j-HLA-DP5, blue lines) was transfected into HEK293T cells. The transfectants were mixed with serum from the representative GD patient (no. 57) or wild-type (WT) K1-18 mAb, and IgG Ab binding was detected. The expression of HLA-DP and TSHR is also shown. (**B** and **D**) (B) Bar plots of (A), and (D) bar plots of (C); the MFIs for three independent cell stains are shown with the mean ± SD, multiple *t* tests. Mock: Empty pME18S plasmid. **P < 0.01, ***P < 0.001, and ****P < 0.0001. (**E**) Schematic representation of TSHR ECD complexed with the peptide-binding groove HLA-DP5 molecule. TRAb binding is shown. All data are representative of at least three independent experiments.

Peptide-binding groove of MHC class II molecules is known to be open at both ends, suggesting that large antigens can bind to the MHC class II molecules (*25*). To further determine the mechanism by which TRAb recognizes TSHR in the presence of HLA-DP5, we generated HLA-DP5 covalently attached with a Japanese cedar (*Cryptomeria japonica*, Cry-j-1) pollen peptide, which is known to bind to the peptide-binding groove of HLA-DP5 (*26*, *27*). As a control, we generated HLA-DP5 covalently attached with an HLA-Cw3 peptide that is preferentially presented on HLA-DR4 (*28*). Autoantibodies from the GD patient and K1-18 mAb did not recognize TSHR in the presence of the Cry-j-1 peptide–attached HLA-DP5 molecules, despite almost identical levels of TSHR expression (Fig. 2, A and B). On the other hand, the HLA-Cw3 peptide did not affect the Ab binding at all (Fig. 2, A and B). TRAb is known to preferentially recognize the cleaved TSHR extracellular domain (ECD), while its recognition of wild-type TSHR is poor; however, the mechanism for this has not been elucidated (*4*, *29*). We generated TSHR ECD and found that TSHR ECD could be expressed on the cell surface in the presence of HLA-DP5 and that it was also recognized by autoantibodies from the GD patient and K1-18 mAb (Fig. 2, C and D). On the other hand, the Cry-j-1 peptide–attached HLA-DP5 molecules, but not the HLA-Cw3 peptide–attached HLA-DP5 molecules, markedly reduced the expression level of TSHR ECD and the binding level of autoantibodies from the GD patient and K1-18 mAb to TSHR ECD (Fig. 2, C and D). These findings suggest that the binding of TSHR ECD to the peptide-binding groove of HLA-DP5 results in structural changes that lead to the exposure of cryptic epitopes for TRAb (Fig. 2E).

### Autoantibodies from GD patient bind to TSHR in an HLA-DP allele–dependent manner

**Fig. 3.**
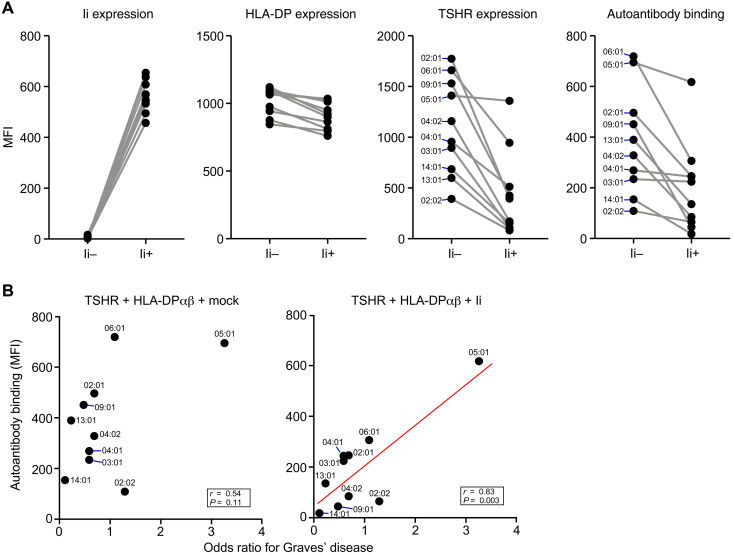
Binding of autoantibodies from GD patient to TSHR is correlated with the odds ratios for GD susceptibility for each HLA-DP allele. (**A**) Human TSHR ECD was cotransfected with each HLA-DPA1/HLA-DPB1 allele in which the HLA-DPB1 alleles are indicated in the figure. Human Ii was also cotransfected (Ii+) or not (Ii−). The MFIs for the expression of Ii, HLA-DP, or TSHR are shown. The MFIs for the binding of autoantibodies from the representative GD patient serum (no. 57) to TSHR ECD complexed with each HLA-DP molecule are also shown. (**B**) The MFIs for the binding of autoantibodies from the representative GD patient serum (no. 57) to TSHR ECD complexed with each HLA-DP molecules in (A) were plotted against the odds ratios for GD susceptibility for each HLA-DP allele (*10*). Linear regression line (red line), correlation coefficient (*r*), and *P* value for the regression line are shown. Each HLA-DPB1 allele was combined with the HLA-DPA1 allele for the highest haplotype frequency. Mock: Empty pME18S plasmid. All data are representative of at least three independent experiments.

The invariant chain (Ii) binds to the peptide-binding groove of newly synthesized MHC class II molecules in the endoplasmic reticulum, thereby preventing MHC class II molecules from binding to peptides or misfolded proteins (*30*). The expression of Ii is regulated by the MHC class II transactivator, and HLA class II–expressing cells basically express Ii (*5*). We investigated the effect of Ii on the association of TSHR with different HLA-DP alleles. In the presence of Ii, the expression levels of TSHR ECD presented by HLA-DP molecules were decreased for most alleles except HLA-DP5. Furthermore, the binding of autoantibodies from the GD patient to TSHR was inhibited in the presence of Ii, except for the HLA-DP5 allele (Fig. 3A). There was a significant correlation between the level of autoantibodies bound to the TSHR ECD/HLA-DP complex and the risk of developing GD conferred by each HLA-DP allele in the presence of Ii (*r* = 0.83; *P* = 0.003), but not in the absence of Ii (*r* = 0.54; *P* = 0.11). This suggests that the formation of the TSHR/HLA-DP complex in the presence of Ii is involved in the etiology of GD (Fig. 3B).

### TSHR peptides presented on HLA-DP5 are not involved in GD autoantibody binding to TSHR/HLA-DP complex

**Fig. 4.**
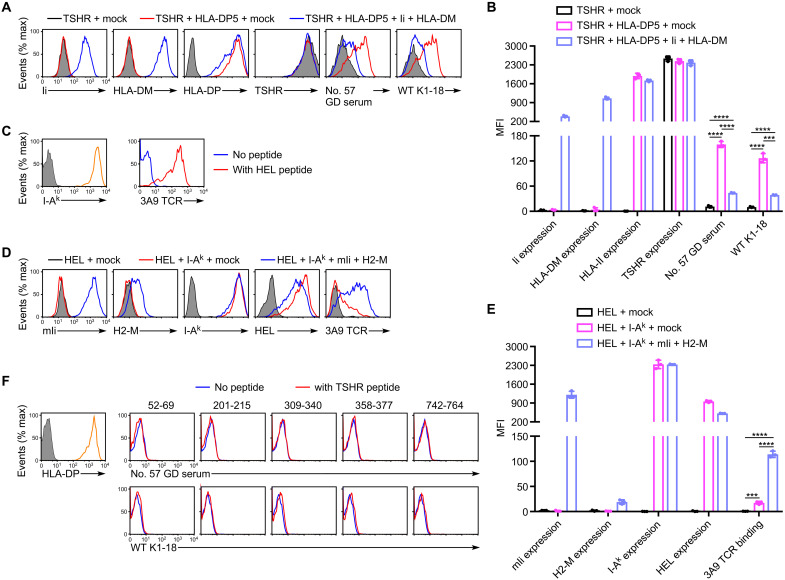
TSHR protein complexed with HLA-DP5, but not peptides, is recognized by autoantibodies from GD patients. (**A**) Full-length human TSHR (TSHR) (shaded histograms), TSHR with HLA-DP5 (red lines), or TSHR with HLA-DP5, human Ii (Ii), and HLA-DM (blue lines) was transfected into HEK293T cells. After 24 hours, the transfectants were mixed with serum from the no. 57 GD patient or wild-type (WT) K1-18 mAb, and IgG Ab binding was detected. The expression of Ii, HLA-DM, HLA-DP, and TSHR is also shown. (**B**) Bar plots of (A); the MFIs for three independent cell stains are shown with the mean ± SD, multiple *t* tests. (**C**) I-A^k^ was transfected into HEK293T cells, and the expression of I-A^k^ is shown (left yellow line). Shaded histogram shows the same cells stained with APC-conjugated anti-mouse IgG Ab only as control. The I-A^k^ transfectants were pulsed with HEL 48–61 peptide (10 μM). The pulsed cells were stained with 3A9 TCR–Fc fusion protein (right). (**D**) Flag-tagged HEL (shaded histograms), HEL with I-A^k^ (red lines), or HEL with I-A^k^, mouse Ii (mIi), and H2-M (blue lines) was transfected into HEK293T cells. After 24 hours, the transfectants were stained with 3A9 TCR–Fc fusion protein. The expression of mIi, H2-M, I-A^k^, and HEL is also shown. (**E**) Bar plots of (D); the MFIs for three independent cell stains are shown with the mean ± SD, multiple *t* tests. (**F**) HLA-DP5 was transfected into HEK293T cells, and the expression of HLA-DP5 is shown (left yellow line). Shaded histogram shows the same cells stained with APC-conjugated anti-mouse IgG Ab only as control. The HLA-DP5 transfectants were pulsed with TSHR peptides (50 μM). The pulsed cells were mixed with serum from the no. 57 GD patient or wild-type (WT) K1-18 mAb, and IgG Ab binding was detected (right). Mock: Empty pME18S plasmid. ***P < 0.001 and ****P < 0.0001. All data are representative of three independent experiments.

Autoantibodies from GD patients preferentially bound to TSHR in the presence of HLA-DP5 molecules. However, there is a possibility that TSHR peptides presented on HLA-DP5 are involved in the autoantibody binding to the TSHR/HLA-DP5 complex. To test this possibility, we transfected TSHR and HLA-DP5 into human embryonic kidney (HEK) 293T cells in the presence or absence of invariant chain (Ii) and HLA-DM that are required for peptide presentation by MHC class II molecules (*6*). Anti-TSHR autoantibodies bound to TSHR in the absence of Ii and HLA-DM. However, the autoantibody binding to TSHR was decreased in the presence of Ii and HLA-DM that promotes peptide presentation by MHC class II molecules (Fig. 4, A and B). This suggested that autoantibodies recognize TSHR protein complexed with HLA class II molecules rather than TSHR peptide presented on MHC class II molecules.

To confirm that peptides are preferentially presented on MHC class II molecules in the presence of Ii and HLA-DM, we analyzed the effect of Ii and HLA-DM on peptide presentation by MHC class II molecules using hen egg lysozyme (HEL) antigen and Fc fusion protein of 3A9 T cell receptor (TCR), a specific TCR to HEL peptide presented on mouse MHC class II molecules, I-A^k^ (*31*). Fc fusion protein of 3A9 TCR specifically recognized I-A^k^ transfectants pulsed with HEL peptide but not peptide nonpulsed I-A^k^ transfectants, indicating that 3A9 TCR–Fc fusion protein specifically detects HEL peptides presented on I-A^k^ molecules (Fig. 4C). 3A9 TCR–Fc fusion protein efficiently bound to HEL and I-A^k^ transfectants in the presence of Ii and H2-M (mouse HLA-DM) but not in the absence of them (Fig. 4, D and E). This suggests that TSHR peptides are also efficiently presented on HLA-DP5 molecules in the presence of Ii and HLA-DM, and that TSHR peptides presented on HLA-DP5 are unlikely to be a target for GD autoantibodies.

To further address the possibility that the TSHR peptide/HLA-DP5 complex is not a target for autoantibodies from GD patients, we analyzed TSHR peptides presented on HLA-DP5 on TSHR and HLA-DP5 transfectants by mass spectrometry and identified five TSHR peptides. As predicted, HLA-DP5 transfectants pulsed with these five TSHR peptides were not recognized by GD autoantibodies at all (Fig. 4F), suggesting that the TSHR peptide/HLA-DP5 complex is unlikely to be the target of GD autoantibodies.

### TSHR forms a complex with MHC class II molecules, which are aberrantly expressed on thyroid follicular cells

**Fig. 5.**
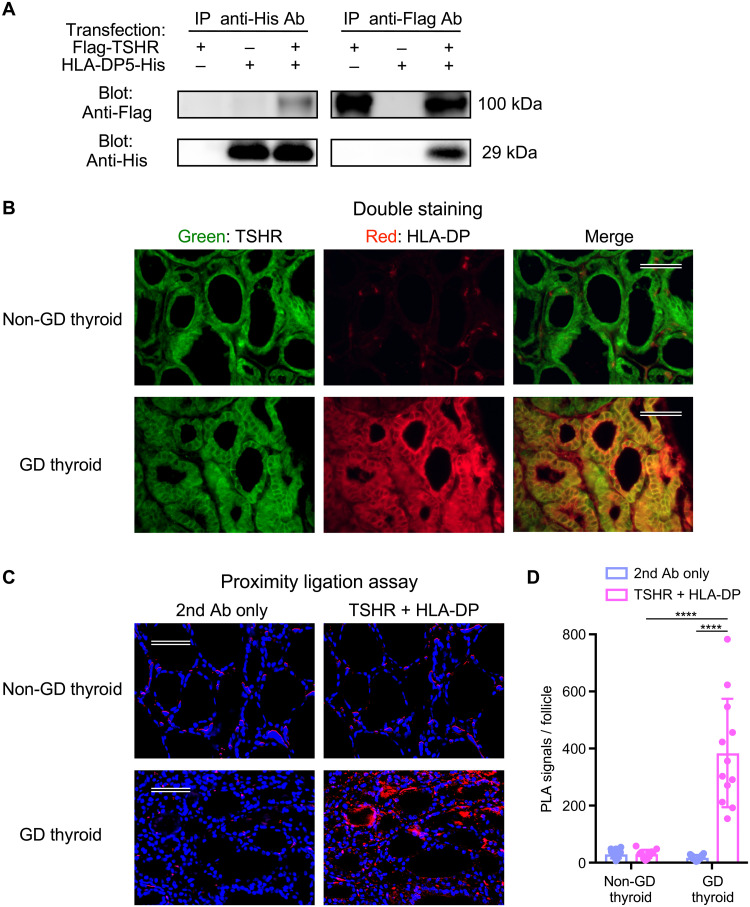
TSHR forms a complex with MHC class II molecules. (**A**) Flag-tagged full-length human TSHR (Flag-TSHR) and His-tagged HLA-DP5 (HLA-DP5-His) were transfected into HEK293T cells. HLA-DP5 or TSHR was immunoprecipitated from the cell lysates and immunoblotted. (**B**) Tissue sections from GD patients (*n* = 2) and non–GD patients (*n* = 2) were stained with anti-TSHR Ab (green) and anti–HLA-DP Ab (red). Scale bars, 50 μm. (**C**) In situ association of TSHR with HLA-DP in tissue sections from GD patients (*n* = 2) and non–GD patients (*n* = 2) was analyzed via PLA. Photographs were taken at the same locations on consecutive tissue sections to compare the difference between the secondary Abs only and anti-TSHR/anti–HLA-DP. PLA signals and nuclei are shown as red and blue colors, respectively. Scale bars, 50 μm. (**D**) Bar plots of (C); the PLA signals per follicle are shown with the mean ± SD, multiple *t* tests, *n* = 12. ****P < 0.0001. Data are mixed for the two patients, respectively. Representative data from at least three independent experiments are shown.

We analyzed cells expressing both TSHR and HLA-DP5 and found that TSHR was coprecipitated with HLA-DP5 (Fig. 5A), suggesting that TSHR forms a complex with HLA-DP5. We then examined whether TSHR/HLA-DP complexes are present as target antigens for autoantibodies in the thyroid tissue of GD patients. We analyzed the expression of TSHR and HLA-DP in thyroid tissues obtained from GD patients via immunofluorescence staining and proximity ligation assays (PLAs), which is used to detect protein-protein interactions closer than 40 nm (*32*). TSHR was localized to both non-GD thyroid and GD thyroid tissues, but HLA-DP was only detected in GD thyroid tissues (Fig. 5B). Furthermore, PLA signals between TSHR and HLA-DP were observed around thyroid follicles in the thyroid tissues from GD patients, but not in the non-GD thyroid tissues (Fig. 5, C and D). These data demonstrate that HLA-DP is aberrantly expressed in the thyroid tissue of GD patients, and that TSHR forms a complex with HLA-DP and is recognized by autoantibodies from GD patients.

### mTSHR complexed with MHC class II molecules abrogates self-tolerance

**Fig. 6.**
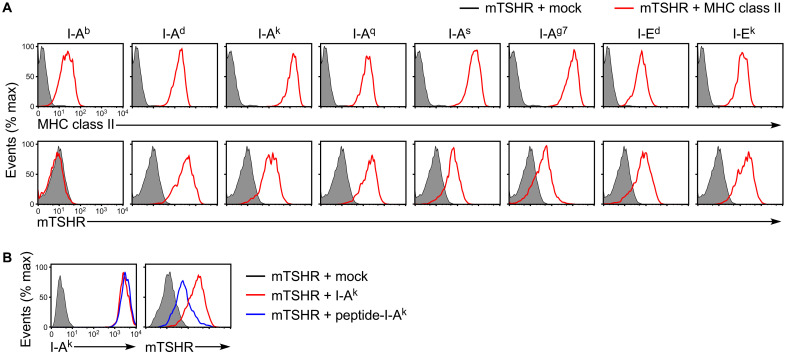
Mouse TSHR ECD is presented on the cell surface by mouse MHC class II molecules. (**A**) Mouse TSHR (mTSHR) ECD (shaded histograms) or mTSHR ECD with each mouse MHC class II allele (red lines) was transfected into HEK293T cells, respectively. The expression of MHC class II and TSHR is shown. (**B**) mTSHR ECD (shaded histograms), mTSHR ECD with I-A^k^ (red lines), or mTSHR ECD with HEL peptide–attached I-A^k^ (peptide-I-A^k^, blue lines) was transfected into HEK293T cells. The expression of I-A^k^ and TSHR is shown. Mock: Empty pME18S plasmid. All data are representative of at least three independent experiments.

Next, we analyzed the in vivo pathogenicity of TSHR complexed with MHC class II molecules in mice. We first analyzed several mouse MHC class II alleles and found that mouse TSHR (mTSHR) ECD can be transported to the cell surface by most of the mouse MHC class II alleles, except I-A^b^ (Fig. 6A and fig. S1). We then selected I-A^k^ for further analysis, although the amino acid homology between I-A^k^ and HLA-DP5 is about 60%. We generated a HEL peptide, which is one of the most common naturally bound peptides in the peptide-binding groove of I-A^k^ (*33*). The surface expression of mTSHR ECD was markedly suppressed in the presence of HEL peptide–attached I-A^k^, suggesting that mTSHR ECD binds to the peptide-binding groove of I-A^k^, similar to the binding of human TSHR to HLA class II molecules (Fig. 6B).

**Fig. 7.**
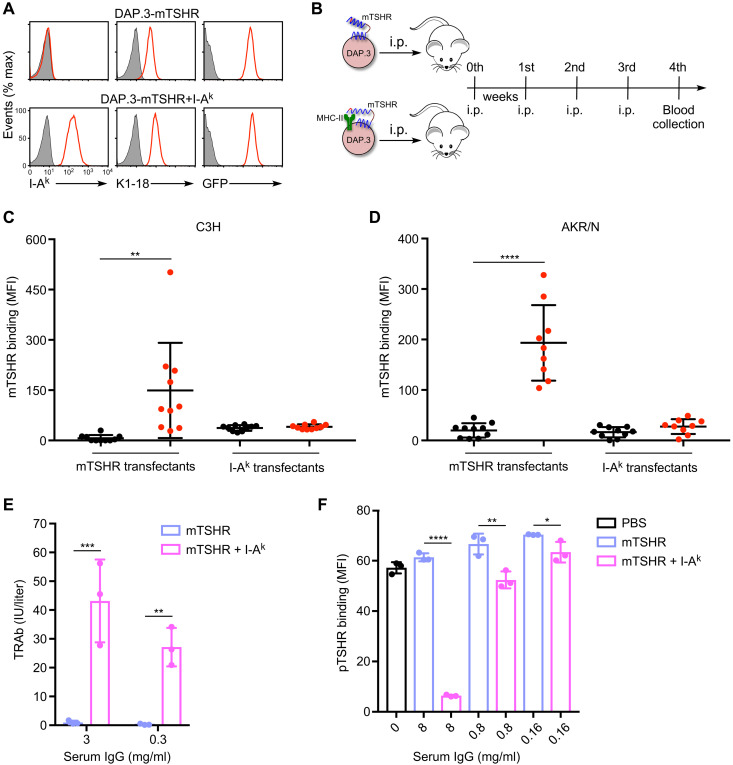
Autoantibodies are induced by mTSHR complexed with MHC class II molecules in mice. (**A**) DAP.3 cells expressing mTSHR alone (first row) or mTSHR and I-A^k^ (second row) were stained with anti–I-A^k^ mAb or K1-18 mAb (red), respectively. The GFP level is also shown (red). Shaded histograms are DAP.3 parental cells. (**B**) Timeline for the administration of DAP.3 transfectants into mice. i.p., intraperitoneally. (**C** and **D**) HEK293T cells transfected with mTSHR or I-A^k^ were mixed with sera from (C) C3H/HeN mice immunized with DAP.3 cells expressing mTSHR alone (black, *n* = 10) or mTSHR and I-A^k^ (red, *n* = 10), or (D) AKR/N mice immunized by DAP.3 cells expressing mTSHR alone (black, *n* = 10) or mTSHR and I-A^k^ (red, *n* = 9). IgG Ab binding to TSHR transfectants was analyzed. The MFIs of Ab binding to mTSHR are represented as dots (one per mouse) with the mean ± SD, unpaired two-tailed Student’s *t* test. (**E**) TRAb in the mice immunized with DAP.3 cells expressing mTSHR alone (green bars) or mTSHR and I-A^k^ (yellow bars). Data are represented as the mean ± SD of triplicate experiments, two-way analysis of variance (ANOVA). (**F**) HEK293T cells transfected with porcine TSHR (pTSHR) were mixed with PBS (gray bar), sera IgGs from mice immunized by DAP.3 cells expressing mTSHR alone (green bars), or mTSHR and I-A^k^ (yellow bars), followed by staining with M22 mAb. The MFIs for the binding of M22 mAb to pTSHR are bar plotted as the mean ± SD of triplicate experiments, unpaired two-tailed Student’s *t* test. All MFIs were calculated by subtracting the MFI of Ab binding to that of GFP-negative transfectants. All data are representative of at least three independent experiments. *P < 0.05, **P < 0.01, ***P < 0.001, and ****P < 0.0001.

We next explored whether TSHR/MHC class II complexes are involved in autoantibody production. Previous studies have reported that TRAbs are induced in mice via immunization with human TSHR (*34*–*38*). However, because human TSHR is a xenoantigen for mice, these mouse models do not adequately represent the pathophysiological condition of GD. Accordingly, we generated a mouse L cell fibroblast clone (DAP.3) expressing mTSHR and I-A^k^ molecules. As a control, I-A^k^ molecules were knocked out using the CRISPR-Cas9 system. TSHR expression levels in mTSHR + I-A^k^– and mTSHR alone–expressing cells were almost the same (Fig. 7A). These cells were injected intraperitoneally into C3H/HeN or AKR/N mice, with I-A^k^, once a week for 4 weeks (Fig. 7B). Mice injected with mTSHR + I-A^k^–expressing cells produced anti-mTSHR autoantibodies, whereas mice injected with cells expressing mTSHR alone did not (Fig. 7, C and D). Control staining revealed that no mice produced anti–I-A^k^ autoantibodies (Fig. 7, C and D). These data suggest that the expression of MHC class II molecules conferred TSHR antigenicity differently from normal TSHR and resulted in autoantibody production against TSHR. Furthermore, autoantibodies were induced in both C3H/HeN and AKR/N mice, which indicates that the immune response to the TSHR/MHC class II complex is not influenced by mouse strain.

To investigate whether the autoantibodies induced in mice were thyroid-stimulating autoantibodies, purified mouse serum immunoglobulin Gs (IgGs) from mice treated with cells expressing TSHR + I-A^k^ or TSHR alone were measured using a third-generation TRAb kit. High titers of TRAb were detected in the IgGs from mice treated with mTSHR + I-A^k^–expressing cells, but not from those treated with cells expressing mTSHR alone (Fig. 7E). Similar results were obtained when the inhibition of M22 mAb binding to TSHR by serum IgGs was analyzed (Fig. 7F). These data demonstrate that autoantibodies that recognize epitopes similar to thyroid-stimulating autoantibodies were induced by TSHR/MHC class II complexes.

### DISCUSSION

Most autoimmune disease animal models rely on xenoantigen immunization to induce an immune response to the self-antigens (*39*); however, immunization with xenoantigens does not reflect pathophysiological conditions. Previously reported animal models of GD have also used human TSHR for immunization (*34*–*38*). Alternatively, TSHR-deficient mice were immunized with mTSHR to induce an Ab response, although this also did not reflect a pathophysiological conditions (*40*). Autoantibody production was observed in normal mice simply by transferring cells expressing both mTSHR and MHC class II to mice. Furthermore, in addition to I-A^k^, other mouse MHC class II alleles such as I-A^d^, I-A^q^, and I-E^k^ were also effective in presenting mTSHR ECD on the cell surface (Fig. 6A), suggesting that mouse MHC class II molecules are less restricted in their expression of TSHR than are HLA class II molecules. Therefore, this model may also be applicable to other mouse strains that have other MHC class II alleles. In conclusion, our findings suggest that TSHR complexed with MHC class II molecules plays an important role in the abrogation of self-tolerance and induction of autoantibody production.

Exposure of cryptic epitopes on self-antigens is proposed to be one of the mechanisms for autoantibody production (*41*). As cryptic autoantibody epitopes are exposed on cleaved TSHR, the cleavage of TSHR has been suggested to be involved in autoantibody production (*4*, *29*). However, TSHR is constitutively cleaved in vivo in healthy individuals (*42*); thus, the production of cleaved TSHR by itself cannot explain the mechanism underlying autoantibody production. We demonstrated that autoantibody epitopes on TSHR ECD are exposed through the association with the peptide-binding groove of MHC class II molecules with GD risk alleles. Furthermore, our results revealed that the TSHR and MHC class II complex was present on thyroid epithelial cells in GD patients, and that anti-TSHR autoantibodies were produced by L cells expressing TSHR and MHC class II molecules. Therefore, cryptic autoantibody epitopes exposed on TSHR through the association with MHC class II molecules are involved in the autoantibody production in GD. As L cells and thyroid cells do not express the costimulatory molecules, such as CD80, required for T cell priming, it is unlikely that L cells expressing MHC class II molecules directly primed autoreactive T cells. MHC class II molecules are reported to be released from cells as extracellular vesicles or soluble molecules (*43*–*46*). Therefore, it is possible that TSHR/MHC class II complexes with exposed cryptic autoantibody epitopes are released from cells and initiate the immune response against the complexes in lymphoid tissues.

In addition to HLA-DP5, other HLA class II alleles such as HLA-DRB1*03:01 (*47*), HLA-DRB1*03:04 (*48*), HLA-DR4 (*49*), and HLA-DRB1*14:03 (*11*) have also been reported to be associated with the risk of GD, suggesting that TSHR complexed with HLA class II molecules with these alleles might also be involved in the production of autoantibodies through the mechanism similar to that of HLA-DP5. Therefore, further studies are required to elucidate the role of these HLA class II molecules in autoantibody production.

Ii binds to newly synthesized MHC class II molecules in the endoplasmic reticulum, thereby preventing MHC class II molecules from binding to peptides or misfolded proteins (*30*). However, the affinity of the Ii to MHC class II molecules is dependent on the alleles of the MHC class II molecules (*50*). Therefore, it is possible that the unfolded peptide-like region on the misfolded proteins binds to the peptide-binding groove of MHC class II molecules instead of Ii in the endoplasmic reticulum. As misfolded proteins bound to MHC class II molecules do not have lysosomal targeting signals, unlike Ii, the proteins bound to MHC class II molecules are directly transported to the cell surface (*13*, *14*). Binding of myeloperoxidase to the MHC class II molecules of specific alleles was observed in primary activated neutrophils (*16*). Although normal tissues do not express MHC class II molecules, MHC class II expression is readily induced in most tissues by cytokines, such as interferon-γ, produced as a result of infection or inflammation (*51*). It has been suggested that certain virus infections are often observed before the onset of autoimmune diseases. Therefore, MHC class II molecules aberrantly induced by viral infection or inflammation seem to form self-antigen/MHC class II complexes that initiate autoantibody production. Further studies to elucidate the mechanism underlying aberrant MHC class II expression in the tissues of patients with autoimmune disease and the mechanism through which the autoimmune response is induced by misfolded self-antigens are important to our understanding of the fundamental mechanism underlying autoimmune diseases.

## MATERIALS AND METHODS

### Human samples

The diagnosis of GD was based on the guidelines of the Japan Thyroid Association. HLA-DPB1 genotypes were determined using the Luminex assay system (Luminex Corporation) with HLA typing kits (Wakunaga). TRAbs were determined by the third-generation assay. HLA-DPB1 alleles and TRAb titers for GD patients are shown in table S1. The collection and use of human sera and thyroid tissues were approved by the Institutional Review Board of Osaka University (24-7) and Kyushu University (417-00). Written informed consent was obtained from all participants according to the relevant guidelines of the institutional review board. Sera from healthy donors were purchased from George King Bio-Medical Inc.

### Plasmids

Complementary DNAs (cDNAs) for different HLA class II alleles, HLA-DMα (DMA*01:02), HLA-DMβ (DMB*01:01), human Ii (NM_004355.2), human TSHR (AY429111.1), human TSHR ECD (acid residues 22 to 282), mTSHR (NM_011648.5), mTSHR ECD (acid residues 22 to 282), porcine TSHR (pTSHR) (NM_214297.1), mouse Ii (NM_010545.3), H2-Mα (U35324.1), H2-Mβ1 (NM_010387.3), I-A^b^α (NM_010378.3), I-A^b^β (NM_207105.3), I-A^d^α (AY452201.1), I-A^d^β (BC010322.1), I-A^k^α (V00832.1), I-A^k^β (M13538.1), I-A^q^α (BC029620.1), I-A^q^β (M13537.1), I-A^s^α (KJ650232.1), I-A^s^β (BC057998.1), I-A^g7^β (AK171211.1), I-Eα (KP012537.1), I-E^d^β (BC132163.1), and I-E^k^β (M36939.1) were cloned from cDNA prepared from the human peripheral blood mononuclear cells of healthy donors, human cell lines, thyroid tissues, and mouse spleen. Some HLA-DP alleles were generated using the QuikChange Multi Mutagenesis Kit (Agilent Technologies) from HLA genes with similar sequences. All cDNA sequences for HLA were based on information contained in the IMGT/HLA Database (https://ebi.ac.uk/ipd/imgt/hla/). HLA-DPA1*01:03/HLA-DPB1*02:01, HLA-DPA1*01:03/HLA-DPB1*02:02, HLA-DPA1*01:03/HLA-DPB1*03:01, HLA-DPA1*01:03/HLA-DPB1*04:01, HLA-DPA1*01:03/HLA-DPB1*04:02, HLA-DPA1*02:02/HLA-DPB1*05:01, HLA-DPA1*01:03/HLA-DPB1*06:01, HLA-DPA1*02:01/HLA-DPB1*09:01, HLA-DPA1*02:01/HLA-DPB1*13:01, and HLA-DPA1*02:01/HLA-DPB1*14:01 were combined so that each group had the highest haplotype frequency, as confirmed by the Allele Frequency Net Database (http://allelefrequencies.net/hla.asp). HLA-DPB1*05:01 containing a covalently attached Cry-j-1 pollen peptide (acid residues 209 to 226, DDKSMKVTVAFNQFGPNC) or HLA-Cw3 peptide (acid residues 25 to 41, GSHSMRYFYTAVSRPGR) and I-A^k^β containing a covalently attached HEL peptide (acid residues 48 to 61, DGSTDYGILQINSR) were generated as previously described (*52*). HLA-DPA1*02:02 plasmid was also C-terminally His-tagged for immunoprecipitation and Western blotting. The human TSHR ECD and mTSHR ECD plasmids contained a BM40 signal sequence, followed by an N-terminal Flag tag for flow cytometry, immunoprecipitation, or Western blotting. A cDNA for the mutant HEL in which two cysteine residues at positions 30 and 64 were replaced with alanine was generated as described previously (*13*). Each cDNA was cloned into the pME18S expression vector. mTSHR, I-A^k^α, and I-A^k^β were also cloned into the pMxs retroviral expression vector. The nucleotide sequence of each cDNA was confirmed by Sanger sequencing (ABI3130xl, Thermo Fisher Scientific).

### 3A9 TCR–Fc fusion protein

cDNAs encoding 3A9 TCR α- and β-chain gene were cloned from 3A9 T cell hybridoma (*31*). 3A9 TCR α-chain V-J region linked with ECD of 3A9 TCR β chain by a GGGGSGGGGSGGGGS linker was cloned into a pME18S expression vector containing Fc segment of human IgG1, as described previously (*53*). Plasmids encoding 3A9 TCR–IgG Fc were transiently transfected into HEK293T cells, and then after 3 days, the culture supernatant was collected for cell staining.

### Antibodies and cell lines

Anti–HLA-DP (HL-38, Sigma-Aldrich), anti–HLA-DP-DQ-DR (WR18, Bio-Rad), anti-rat RT1B (OX-6, BD Biosciences), anti–MHC class II (M5/114.15.2, eBioscience), anti-TSHR (2C11, Santa Cruz Biotechnology), anti-His (9F2, Wako), and anti-Flag (purified IgG, M2, Sigma-Aldrich) Abs were used for flow cytometry. Anti-Flag (biotin-conjugated, M2, Sigma-Aldrich) and anti-His (28-75, Wako) Abs were used for immunoprecipitation. Anti-Flag (purified IgG, M2, Sigma-Aldrich) and anti-His (9F2, Wako) Abs were used for Western blotting. Anti–HLA-DP (E-20, Santa Cruz Biotechnology) and anti-TSHR (HPA026680, Sigma-Aldrich) polyclonal Abs were used for immunofluorescence staining and PLA. To produce human anti-TSHR mAbs, the V regions of the mAbs were synthesized according to the published sequence (accession numbers: M22 heavy chain, 3G04_B; λ chain, 3G04_A; K1-18 heavy chain, AMF37397; λ chain, AMF37401; K1-70 heavy chain, 2XWT_A; and λ chain, 2XWT_B). For germlined K1-18, some mutants were generated using the QuikChange Multi Mutagenesis Kit (Agilent Technologies) from wild-type K1-18 V regions (heavy chain, N31S, Y54G, and V97A; λ chain, N31S and N32S). These V regions were cloned into pME18S expression vectors containing the secreted form of IgG1 constant region as previously described (*14*). HEK293T cells were purchased from RIKEN Cell Bank.

### Animals

Six-week-old female C3H/HeN mice and AKR/N mice were purchased from Japan SLC. Animals were maintained under specific pathogen–free conditions. All the experiments were conducted according to the guidelines for the Animal Research Committee of the Research Institute for Microbial Diseases, Osaka University.

### Flow cytometry

Cells were incubated with mAbs (anti–HLA-DP, anti–HLA-DP-DQ-DR, anti-rat RT1B, anti–MHC class II, anti-TSHR, anti-His, and anti-Flag) followed by allophycocyanin (APC)–conjugated anti-mouse or anti-rat IgG Ab (Jackson ImmunoResearch). For the analysis of human anti-TSHR mAbs, the cells were incubated with M22, K1-18, K1-70, and germlined K1-18, followed by APC-conjugated anti-mouse or anti-human IgG Ab (Jackson ImmunoResearch). Stained cells were analyzed using FACSCalibur (Becton Dickinson). For intracellular staining, cells were fixed and permeabilized with a fixation/permeabilization solution (BD Biosciences). Intracellular Ii, HLA-DM, and H2-M were detected with anti-human Ii (LN2, BD Biosciences), anti-mouse Ii (In1, BioLegend), anti–HLA-DM (MaP.DM1, Santa Cruz Biotechnology), and anti-mouse H2-M (2E5A, BD Biosciences), followed by APC-conjugated anti-mouse or anti-rat IgG Ab. 3A9 TCR–Fc fusion protein was mixed with APC-conjugated anti-human IgG Ab before adding to cells to increase the affinity. All mean fluorescence intensities (MFIs) were calculated by subtracting the MFI of Ab binding to that of green fluorescent protein (GFP)–negative transfectants.

### Analysis of autoantibody binding to the TSHR/HLA-DP complex

Human TSHR was cotransfected together with HLA-DPαβ and GFP into HEK293T cells. The transfectants were mixed with sera (1:200 dilution) from patients with GD, followed by APC-conjugated anti-human IgG Ab (Jackson ImmunoResearch). Stained cells were analyzed using FACSCalibur. The MFIs of autoantibody binding to TSHR- and HLA-DP–transfected cells were calculated by subtracting the MFI of autoantibody binding to that of GFP-negative transfectants.

### TSHR peptide elution

TSHR peptides were eluted from TSHR and HLA-DP5–expressing cells according to the protocol as previously described (*54*). Briefly, human TSHR was cotransfected together with C-terminal His-tagged HLA-DPA1*02:02 and HLA-DPB1*05:01 (HLA-DP5-His) into HEK293T cells. After 48 hours, transfected cells were lysed in lysis buffer [10 mM tris and 150 mM NaCl (pH 7.5)] containing 0.5% NP-40 (Sigma-Aldrich). HLA-DP5-His was precipitated with anti-His mAb (28-75) and protein G–Sepharose (GE Healthcare) and then eluted with 10% acetic acid. The acid-extracted peptides were filtered with a 10,000 molecular weight cutoff membrane filter (Sartorius Vivaspin) and then analyzed by mass spectrometry. Five HLA-DP5–matched TSHR peptides were finally identified (52–69, PSTQTLKLIETHLRTIPS; 201–215, KLDAVYLNKNKYLTV; 309–340, LRQRKSVNALNSPLHQEYEENLGDSIVGYKEK; 358–377, EQEDEIIGFGQELKNPQEET; and 742–764, IENSHLTPKKQGQISEEYMQTVL) using PEAKS software (Bioinformatics Solutions Inc.). These five TSHR peptides were synthesized by GenScript.

### Peptide pulse assay

HLA-DPA1*02:02/HLA-DPB1*05:01 (for TSHR peptide pulse) or I-A^k^α/I-A^k^β (for HEL peptide pulse) was cotransfected together with GFP into HEK293T cells. After 24 hours, transfected cells were divided into 96-well flat-bottom plates (5.0 × 10^4^ cells per well), followed by adding TSHR peptides (50 μM) or HEL peptide (48–61, DGSTDYGILQINSR, 10 μM). Twenty-four hours later, the TSHR peptide–pulsed cells were stained with serum from the GD patient or K1-18 mAb, followed by APC-conjugated anti-human IgG Ab (Jackson ImmunoResearch). The HEL peptide–pulsed cells were stained with 3A9 TCR–Fc fusion protein premixed with APC-conjugated anti-human IgG Ab. The stained cells were analyzed using FACSCalibur.

### Immunoprecipitation and immunoblotting

Cells were lysed in lysis buffer [10 mM tris and 150 mM NaCl (pH 7.5)] containing 0.5% NP-40 (Sigma-Aldrich). HLA-DP5-His and Flag-TSHR were precipitated with anti-His (28-75) mAb and protein G–Sepharose (GE Healthcare) or biotin–anti-Flag (M2) and streptavidin-Sepharose (GE Healthcare), respectively. The immunoprecipitates were eluted by boiling in SDS–polyacrylamide gel electrophoresis sample buffer, separated on 5 to 20% (w/v) polyacrylamide gels (Atto), and transferred onto polyvinylidene difluoride membranes (Millipore). The membranes were incubated with anti-Flag mAb (M2) or anti-His (9F2) Abs, followed by horseradish peroxidase–conjugated anti-mouse IgG (Jackson ImmunoResearch) Ab. Peroxidase activity was detected using the SuperSignal reagent (Thermo Fisher Scientific).

### Immunofluorescence stain

Paraffin-embedded tissue sections from GD patients (CS802478 and CS809728) and individuals without GD (CS804207 and CS811532) were purchased from OriGene. These tissue sections were stained with anti–HLA-DP (E-20, Santa Cruz Biotechnology) and anti-TSHR (HPA026680, Sigma-Aldrich) polyclonal Abs, followed by Alexa Fluor 647–conjugated anti-goat IgG or Alexa Fluor 488–conjugated anti-rabbit IgG Abs (Molecular Probes). The stained tissue sections were analyzed using an Axioplan 2 fluorescence microscope (Zeiss).

### In situ PLA

A Duolink in situ PLA kit was used according to the manufacturer’s instructions (Sigma-Aldrich) for in situ PLAs. Paraffin-embedded tissue sections from the same patients as used in immunofluorescence stain were incubated with anti–HLA-DP (E-20, Santa Cruz Biotechnology) and anti-TSHR (HPA026680, Sigma-Aldrich) polyclonal Abs, and red fluorescent protein PLA signals were developed using anti-goat MINUS and anti-rabbit PLUS PLA probes. Nuclei were stained with 4′,6-diamidino-2-phenylindole fluorescence dye. The assayed tissue sections were analyzed with an Olympus IX83 inverted microscope. The numbers of fluorescent spots from PLA signals per follicle were quantified using ImageJ software.

### Induction of TRAb in mice

DAP.3 cells were stably transfected with full-length mTSHR, followed by internal ribosomal entry site (IRES)–GFP and mouse MHC class II molecules (I-A^k^α/I-A^k^β) using a retroviral transfection system. GFP- and I-A^k^–positive cells were then sorted with a Sony SH800 cell sorter, and stable transfectants were cloned by limiting dilution. Next, I-A^k^ were knocked out using the CRISPR-Cas9 system. Mice were then immunized with mTSHR/I-A^k^ double-positive or mTSHR-positive cells. Briefly, 5 × 10^6^ cells in 1 ml of phosphate-buffered saline (PBS) were injected intraperitoneally once per week. After four injections, blood was drawn, and then the mTSHR- and GFP-transfected HEK293T cells or the I-A^k^α–, I-A^k^β–, and GFP-transfected HEK293T cells were mixed with sera (1:50 dilution), followed by APC-conjugated anti-mouse IgG Ab (Jackson ImmunoResearch). Stained cells were analyzed using FACSCalibur, and the MFIs of TRAb binding to mTSHR- or I-A^k^–transfected cells were calculated by subtracting the MFI of TRAb binding from that of GFP-negative transfectants.

### Third-generation assay for TRAb

Sera from mice immunized by DAP.3 cells expressing mTSHR-IRES-GFP alone or mTSHR-IRES-GFP and I-A^k^ were pooled and purified by protein A–Sepharose using a Profinia protein purification system (Bio-Rad). Purified mouse IgGs were then measured using a third-generation TRAb kit (Lumipulse Presto, Fujirebio Inc.), and chemiluminescence was measured using TriStar LB941 (Berthold Technologies).

### Blocking assay of M22 mAb to TSHR

pTSHR- and GFP-transfected HEK293T cells were mixed with PBS or purified mouse sera IgGs. After washing away free IgGs, the transfectants were stained with M22 mAb, followed by APC-conjugated anti-human IgG Ab (Jackson ImmunoResearch). Stained cells were analyzed using FACSCalibur. The MFIs of M22 binding to pTSHR-transfected cells were calculated by subtracting the MFI of M22 binding to that of GFP-negative transfectants.

### Statistical analysis

Prism 7.0 software (GraphPad) was used for statistical analysis. The details of the statistical tests carried out are indicated in the respective figure legends. *P* values were considered statistically significant if *P* < 0.05. The error bars in the figures represent the SD. Mice were randomized into different groups before experiments.

## Notes

View/request a protocol for this paper from *Bio-protocol*.
