# Supplementary material for: Abrogation of self-tolerance by misfolded self-antigens complexed with MHC class II molecules
Source: Sci Adv. 2022 Mar 4;8(9):eabj9867. doi: 10.1126/sciadv.abj9867 (PMC8896794; doi:10.1126/sciadv.abj9867)
Supplement: Supplementary file 1 — Fig. S1 [file sciadv.abj9867_sm.pdf]

Science Advances
AAAS

Supplementary Materials for

Abrogation of self-tolerance by misfolded self-antigens complexed with MHC class II molecules

Hui Jin, Kazuki Kishida, Noriko Arase, Sumiko Matsuoka, Wataru Nakai, Masako Kohyama, Tadahiro Suenaga, Ken Yamamoto, Takehiko Sasazuki, Hisashi Arase*

*Corresponding author. Email: arase@biken.osaka-u.ac.jp

Published 4 March 2022, *Sci. Adv.* **8**, eabj9867 (2022)
DOI: 10.1126/sciadv.abj9867

The PDF file includes:

Fig. S1
Legend for table S1

Other Supplementary Material for this manuscript includes the following:

Table S1

Supplemental Figure 1

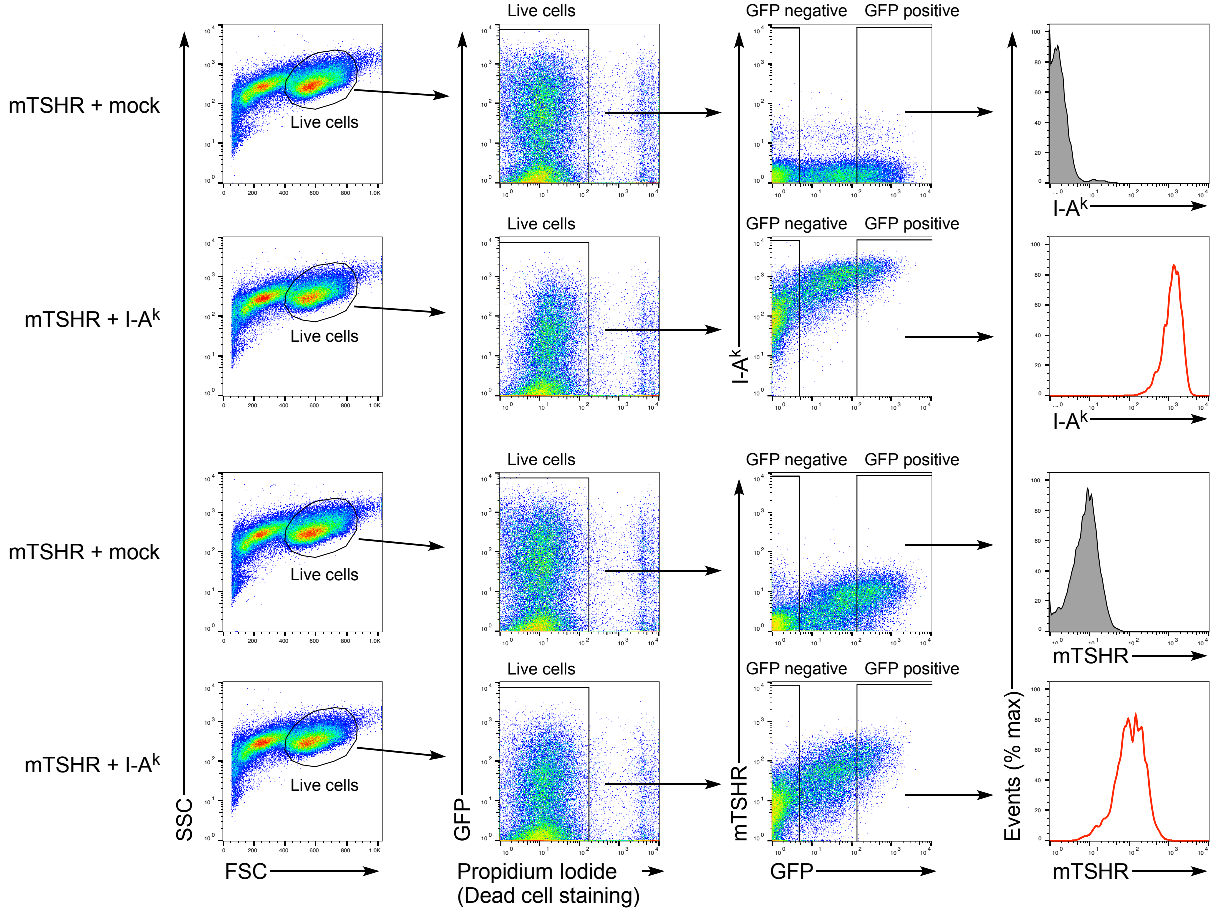

Supplemental Figure 1. Gating strategy for flow cytometry analysis

Representative gating strategy. mTSHR ECD or mTSHR ECD with I-A^k were transfected into HEK293T cells, respectively. The expression of I-A^k and mTSHR are shown. Mock: empty pME18S plasmid.

Supplemental Table 1 (separate file). Information of Graves' disease patients and healthy donors.
